# Genome Sequence of *Vibrio parahaemolyticus* VP152 Strain Isolated from *Penaeus indicus* in Malaysia

**DOI:** 10.3389/fmicb.2016.01410

**Published:** 2016-09-07

**Authors:** Vengadesh Letchumanan, Hooi-Leng Ser, Wen-Si Tan, Nurul-Syakima Ab Mutalib, Bey-Hing Goh, Kok-Gan Chan, Learn-Han Lee

**Affiliations:** ^1^Division of Genetics and Molecular Biology, Institute of Biological Sciences, Faculty of Science, University of Malaya, Kuala LumpurMalaysia; ^2^Novel Bacteria and Drug Discovery Research Group, School of Pharmacy, Monash University Malaysia, Bandar SunwayMalaysia; ^3^UKM Medical Molecular Biology Institute, UKM Medical Centre, Universiti Kebangsaan Malaysia, Kuala LumpurMalaysia; ^4^Center of Health Outcomes Research and Therapeutic Safety, School of Pharmaceutical Sciences, University of Phayao, PhayaoThailand

**Keywords:** *Vibrio parahaemolyticus*, seafood, *Penaeus indicus*, antibiotic resistance, genome

## Introduction

*Vibrio parahaemolyticus* is a Gram-negative bacterium that naturally occurs in marine associated aquatic environments (Letchumanan et al., 2014; Malcolm et al., 2015). This bacterium causes highest number of seafood-associated gastroenteritis in many countries including United States and Asian countries (Scallan et al., 2011; Newton et al., 2012). *V. parahaemolyticus* is often been isolated from aquatic environments such as seawater and marine sediment, as well as from vertebrate and invertebrate seafood products (Shen et al., 2009). The most likely route of infection in humans is reported to be associated with consumption of raw or improperly cooked seafood (Daniels et al., 2000; Jun et al., 2014; Hazen et al., 2015; Raghunath, 2015; Law et al., 2015).

Recently, *V. parahaemolyticus* has been demonstrated to be a major source of infection in the aquaculture industry (Letchumanan et al., 2014; Soto-Rodriguez et al., 2015; Tey et al., 2015). Aquaculture farmers rely on a wide range of antibiotics to prevent (prophylactic use) and treat (therapeutic use) bacterial infections in fish and invertebrates (Cabello et al., 2013). The extensive use of antibiotics and other chemotherapeutics in aquaculture has led to the emergence of multidrug resistant strains in the biosphere (Letchumanan et al., 2015a, 2016; Rao and Lalitha, 2015). Multidrug resistant *V. parahaemolyticus* strains have been isolated and detected from shrimp in Thailand (Yano et al., 2014), Malaysia (Al-Othrubi et al., 2011; Sani et al., 2013; Letchumanan et al., 2015b,c) and China (Peng et al., 2010; Xu et al., 2014). Resistance toward clinically used antibiotics will eventually hamper the treatment of bacterial infections in humans and potentially increase the fatality rate (Daniels et al., 2000). Therefore, monitoring *Vibrio* species in aquaculture surroundings is crucial for both human health and the aquaculture industry.

In our previous study, we have isolated environmental *V. parahaemolyticus* strains from two types of Malaysian shrimp, *Penaeus indicus* and *Solenocera subnuda*. We detected the thermostable direct hemolysin (*tdh*) and thermostable direct related hemolysin (*trh*) virulence genes through a PCR based assay and studied the antibiotic resistance profile of all the isolated strains (Letchumanan et al., 2015c). *V. parahaemolyticus* VP152 was isolated from *Penaeus indicus* (Banana prawn) and originated from a supermarket sample. This strain did not possess both the *tdh* and *trh* virulence genes, which are responsible for causing diseases in humans and marine animals. Despite the fact that *V. parahaemolyticus* VP152 strain does not have *tdh* and *trh* virulence genes properties, the strain cannot be ignored in light of the fact that it exhibits multidrug resistance profiles toward 11/14 antibiotics tested. Based on the antibiotic susceptibility phenotype, the strain exhibited multiple-antibiotic resistance toward ampicillin, oxytetracycline, nalidixic acid, ampicillin/sulbactam, tetracycline, third generation cephalos porins (cefotaxime and ceftazidime), aminoglycosides (amikacin, kanamycin, and gentamicin) and trimethoprim/sulfameth oxazole (Letchumanan et al., 2015c).

This is a worrying situation as the antibiotic resistant profiles shown by *V. parahaemolyticus* VP152 include the recommended antimicrobial agents used in treatment of *Vibrio* spp. infections, including third generation cephalosporin, fluoroquinolones, aminoglycosides, tetracycline, gentamicin, trimethoprim/sulfamethoxazole (Daniels and Shafaie, 2000; Shaw et al., 2014). Therefore, the whole genome sequence of *V. parahaemolyticus* VP152 was studied with respect to the multidrug resistance profiles to gain a better understanding of the antibiotic resistant patterns. The availability of this genome sequence of *V. parahaemolyticus* VP152 will aid as a basis for further in-depth analysis of the antibiotic resistance profile of environmental *V. parahaemolyticus*.

## Materials and Methods

### Genome Sequencing and Assembly

Genomic DNA of VP152 strain was extracted using Masterpure^TM^ DNA purification kit (Epicenter, Illumina Inc, Madison, WI, USA) and subjected to RNase (Qiagen, USA) treatment (Ser et al., 2015). The DNA quality was quantified using NanoDrop spectrophotometer (Thermo Scientific, Waltham, MA, USA), and a Qubit version 2.0 fluorometer (Life Technologies, Carlsbad, CA, USA). Illumina sequencing library of genomic DNA was prepared using Nextera^TM^ DNA Sample Preparation kit (Illumina, San Diego, CA, USA) and library quality was validated by a Bioanalyzer 2100 high sensitivity DNA kit (Agilent Technologies, Palo Alto, CA, USA) prior to sequencing. The genome of VP152 strain was sequenced on MiSeq platform with MiSeq Reagent Kit 2 (2 × 250 bp; Illumina Inc, San Diego, CA, USA). The trimmed sequences were *de novo* assembled with CLC Genomic Workbench version 5.1 (CLC Bio, Denmark).

### Genome Annotation

Gene prediction was carried out using Prodigal 2.6, while rRNA and tRNA were analyzed using RNAmmer and tRNAscan SE version 1.21 (Lowe and Eddy, 1997; Lagesen et al., 2007; Hyatt et al., 2010). Gene prediction and annotation were performed using Rapid Annotation Search Tool (RAST; Aziz et al., 2008). Antibiotic resistance genes were analyzed using antibiotic resistance genes-ANNOTation (ARG-ANNOT; Gupta et al., 2014).

## Results

### Genome Characteristics

The genome of *V. parahaemolyticus* VP152 consists of 4,982,021 bp with mean genome coverage of 183.46-fold and with an average G+C content of 53.4% (**Table 1**). A total of 4809 genes was predicted of which 4638 were identified as protein coding genes. There are 91 RNA genes consisting of 11 rRNAs and 80 tRNAs.

**Table 1:** Comparison of genome sequence of *Vibrio parahaemolyticus* VP152 with other genome sequences.

	*Vibrio parahaemolyticus* VP152	*Vibrio parahaemolyticus* VP551	*Vibrio parahaemolyticus* M0605	*Vibrio parahaemolyticus* AQ4037
Source of isolation	Shrimp	Water source	Environmental	Shrimp
Genome size (bp)	4,982,021	5,226,872	5,429,407	4,939,804
Genome coverage (fold)	183.46	256.00	20.00	7.37
Contig N_50_ (bp)	566,732	712,378	121,988	67,710
Sequencing technology	Illumina MiSeq	SOLiD	Ion Torrent	Sanger
KEGG categories, number of genes (genome %)	61 (1.91)	49 (1.73)	46 (1.71)	49 (1.71)
Cationic antimicrobial peptide (CAMP) resistance, number of genes	36	21	23	20
Vancomycin resistance, number of genes	8	7	7	7
β-Lactam resistance, number of genes	20	27	22	28

### Virulence and Antimicrobial Resistance Genes

**FIGURE 1:**
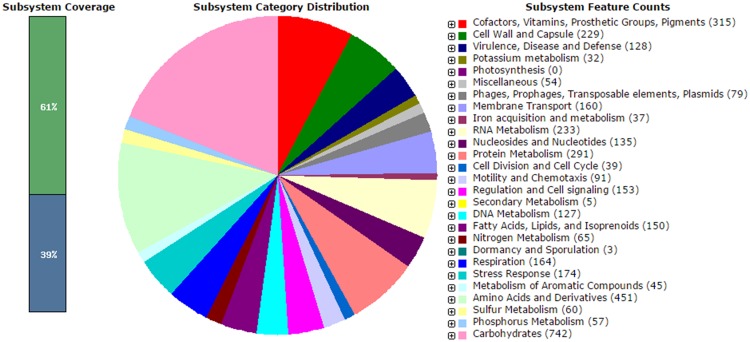
**Subsystem category distribution of *Vibrio parahaemolyticus* VP152 (based on RAST annotation server)**.

The analysis obtained from RAST server revealed 573 subsystems (**Figure 1**). The annotated genome has 97 genes responsible for resistance to antibiotic and toxic compounds including seven genes for mdtABCD multidrug resistance cluster, 19 genes for multidrug resistance eﬄux pumps, four genes for β-lactamase and two genes aminoglycoside adenylyltransferases. The genome sequence of *V. parahaemolyticus* VP152 was compared with three environmental *V. parahaemolyticus* strains, in order to delineate the similarities between the four strains. The genome size of *V. parahaemolyticus* VP152 was similar to strains of *V. parahaemolyticus* and contained several antibiotic resistance genes as shown in **Table 1**. Also, further comparison of hemolysin genes present in *V. parahaemolyticus* VP152 and the selected strains revealed no significant differences.

The genome analysis on ARG-ANNOT noted the presences of tetracycline resistant gene, *Tet* and *Tet-2* gene within the genome. The presence of these genes is closely related to the phenotypic resistance shown by the strain toward oxytetracycline and tetracycline. Furthermore, β-lactam resistance-related gene, *bla* gene of VP152 exhibited 99% similarities when compared to other *V. parahaemolyticus* strain and *Vibrio* species. The phenotypic resistance shown by *V. parahaemolyticus* VP152 toward ampicillin, ampicillin/sulbactam, cefotaxime and ceftazidime is closely related to the gene coding β-lactamase in the genome. The gene coding aminoglycosides adenylyltransferase of *V. parahaemolyticus* VP152 confers resistance phenotype observed toward amikacin, kanamycin, and gentamicin. Based on the annotation tools and detailed analysis of *V. parahaemolyticus* VP152 genome using PlasmidFinder, the genome of *V. parahaemolyticus* VP152 did not recover any plasmid sequence. Even though these genes were commonly found in plasmids, some of the *Vibrio* species including *V. coralliilyticus* and *V. alginolyticus* carry these genes in their chromosomes (Costa et al., 2015). Therefore, the resistant genes observed in *V. parahaemolyticus* VP152 are chromosome mediated.

The multidrug resistance profile seen in the phenotype and genes of *V. parahaemolyticus* VP152 genome illustrates how extensive antibiotics have been utilized in the aquaculture industry. The resistance phenotype observed in this strain could be triggered by the extensive use of permitted antibiotics in the Asian aquaculture industry namely oxytetracycline, tetracycline, quinolone, sulphonamides, and trimethoprim (Rico et al., 2012; Yano et al., 2014). The resistance toward third generation cephalosporins seen in *V. parahaemolyticus* VP152 would further hamper the treatment of *Vibrio* species infection in future. This situation is cause for concern, and warrants more stringent surveillance in the use of antibiotics, as well as the resultant antibiotic resistance in clinically important bacterial species. In summary, the whole genome sequence of *V. parahaemolyticus* VP152 will be useful in future studies to determine antimicrobial resistance and virulence attributes as well as mechanisms that enhance its environmental or host fitness.

### Nucleotide Sequence Accession Numbers

This genome sequence data of VP152 strain sequenced under this study has been deposited in DDBJ/EMBL/GenBank under Accession No. LCUL00000000. The version described in this paper is the first version, LCUL01000000. The genome sequences data are available in FASTA, annotated GenBank flat file, graphical and ASN.1 formats.

## Author Contributions

The experiments, data analysis and manuscript writing were performed by VL and H-LS, while W-ST, N-SA, B-HG, K-GC, and L-HL provided vital guidance, technical support, and proofreading for the work. The research project was founded by L-HL.

## Conflict of Interest Statement

The authors declare that the research was conducted in the absence of any commercial or financial relationships that could be construed as a potential conflict of interest.
